# A *Saccharomyces cerevisiae* Wine Strain Inhibits Growth and Decreases Ochratoxin A Biosynthesis by *Aspergillus carbonarius* and *Aspergillus ochraceus*

**DOI:** 10.3390/toxins4121468

**Published:** 2012-12-10

**Authors:** Loredana Cubaiu, Hamid Abbas, Alan D. W. Dobson, Marilena Budroni, Quirico Migheli

**Affiliations:** 1 Dipartimento di Agraria, Università degli Studi di Sassari, Viale Italia 39, Sassari I-07100, Italy; E-Mails: lcubaiu@uniss.it (L.C.); mbudroni@uniss.it (M.B.); 2 Microbiology Department, University College, Cork, Ireland; E-Mails: hamidnikond70@gmail.com (H.A.); a.dobson@ucc.ie (A.D.W.D.); 3 Centro interdisciplinare per lo sviluppo della ricerca biotecnologica e per lo studio della biodiversità della Sardegna e dell’area mediterranea, Università degli Studi di Sassari, Viale Italia 39, Sassari I-07100, Italy

**Keywords:** polyketide synthase, grape, Ochratoxin A, biological control, *Saccharomyces cerevisiae*, *Aspergillus carbonarius*, *Aspergillus ochraceus*

## Abstract

The aim of this study was to select wine yeast strains as biocontrol agents against fungal contaminants responsible for the accumulation of ochratoxin A (OTA) in grape and wine and to dissect the mechanism of OTA detoxification by a *Saccharomyces cerevisiae* strain (DISAABA1182), which had previously been reported to reduce OTA in a synthetic must. All of the yeast strains tested displayed an ability to inhibit the growth of *Aspergillus carbonarius* both *in vivo* and *in vitro* and addition of culture filtrates from the tested isolates led to complete inhibition of OTA production. *S. cerevisiae* DISAABA1182 was selected and further tested for its capacity to inhibit OTA production and *pks* (polyketide synthase) transcription in *A. carbonarius* and *Aspergillus ochraceus in vitro*. In order to dissect the mechanism of OTA detoxification, each of these two fungi was co-cultured with living yeast cells exposed to yeast crude or to autoclaved supernatant: *S. cerevisiae* DISAABA1182 was found to inhibit mycelial growth and OTA production in both *Aspergilli* when co-cultured in the OTA-inducing YES medium. Moreover, a decrease in *pks* transcription was observed in the presence of living cells of *S. cerevisiae* DISAABA1182 or its supernatant, while no effects were observed on transcription of either of the constitutively expressed calmodulin and β-tubulin genes. This suggests that transcriptional regulation of OTA biosynthetic genes takes place during the interaction between DISAABA1182 and OTA-producing *Aspergilli*.

## 1. Introduction

Ochratoxin A (OTA) is a pentaketide mycotoxin which is produced by several fungal species from the *Aspergillus* and *Penicillium* genera. OTA is nephrotoxic, hepatotoxic, teratogenic and carcinogenic to animals and has been classified by the International Agency of Research on Cancer (IARC) as a possible human renal carcinogen (group 2B) [1]. It is known to occur in a variety of foods and different plant products, including cereals, coffee, cocoa, beer, and wine.

OTA was first detected in wine by Zimmerli and Dick [2], where its presence is commonly ascribed to infection of wine grapes by *Aspergillus carbonarius* and some strains of *Aspergilli* section *nigri*. Currently, maximum permitted levels of 2 μg·kg^−1^ have been established for OTA in wines and grape must-based drinks in the European Union (Commission regulation No. 123/2005 amending Regulation No. 446/2001).

From an epidemiological standpoint, infection by *Aspergillus* spp. immediately prior to or during harvesting, transportation and storage of grapes is considered to be a critical point in OTA contamination of wine [3]. Fungicide treatments at either the pre-harvest or post-harvest stage are however not practical, due to the risk of contamination of the wine by toxic residues [4].

An alternative strategy which has been proposed is the use of biological control based methodologies, which have been proposed as an environmentally sound approach to prevent infection caused by OTA-producing fungi [5]. OTA levels have also previously been shown to decrease during wine fermentations, and this decrease is known to be related to the action of both lactic acid bacteria and yeasts [6,7,8,9,10].

Among the microorganisms with potential utility in biological control-based strategies, yeasts appear particularly promising, particularly due to their ability to colonise plant surfaces or wounds for long periods under dry conditions coupled with an ability to display multiple mechanisms of antagonism [11]. However, the establishment of an antagonistic yeast may be difficult to achieve under unfavourable environmental conditions such as those that they are likely to encounter in the fermenting must, due to the rapid increase in ethanol concentration which takes place, thereby potentially reducing their ability to compete with the resident microbial flora.

We report here on the *in vivo* and *in vitro* biocontrol potential of five *S. cerevisiae* and of two *Kloeckera apiculata* wine strains, which were preliminarily tested against *A. carbonarius*, the main producer of OTA in grape. Moreover, we have evaluated the effect of both living cells and culture filtrates of *S. cerevisiae* DISAABA1182 both on fungal growth and on transcription of the OTA polyketide synthase (*pks*) gene, which is responsible for the biosynthesis of OTA by *A. carbonarius* and *A. ochraceus*.

## 2. Results and Discussion

The need to develop natural alternatives to chemical control strategies has led to the application of various yeast strains as biocontrol agents against various plant pathogenic fungi. There are a large number of reports in the literature detailing that antagonistic yeasts possess effective mechanisms for the control of spoilage microorganisms. These antagonistic properties have been well studied and have been successfully exploited in the biological control of postharvest diseases of fruits [12]. In addition, yeasts have a long history of proven safe use as fermentative starters in food and beverages [13,14,15]. The aims of this study were: (i) to select wine yeast strains which display antagonistic activity against OTA*-*producing *Aspergilli*; and (ii) to investigate possible mechanisms of action of an antagonistic strain of *Saccharomyces cerevisiae* against both *A. carbonarius* and *A. ochraceus*.

During the wine making process, OTA levels are known to decrease, an effect which is believed to be mediated by the activity of the resident microbial flora, particularly of lactic acid bacteria and yeasts [10,16,17,18]. The reduction in OTA level which is mediated by yeasts has been ascribed to different mechanisms including adsorption onto the yeast cell surface, or due to interactions with yeast metabolites. Moreover, Angioni and co-workers have reported that OTA residues do not affect the fermentative process and that reduction of OTA content is a strain-related peculiarity [6].

In the present work, wine strains of *S. cerevisiae* (five strains) and *K. apiculata* (two strains) were tested to assess their potential antagonistic effects against *A. carbonarius* in a co-inoculation assay performed *in vitro* on agar plates using different culture media. This assay was employed in order to select yeast strains that would be able to inhibit the co-inoculated fungus while colonising a common ecological niche. All seven yeast strains displayed an ability to inhibit fungal growth when co-cultured in CYA and YES media, with levels of inhibition in growth of up to 65% being observed. The yeast strain which exhibited the maximal levels of growth inhibition of *A. carbonarius* was *S. cerevisiae* DISAABA1182 following growth on YES medium (Table 1).

When *A. carbonarius* was cultured alone in liquid media, higher levels of OTA were observed from cultures grown on CYB (0.3 ± 0.1 μg/mL) than on YES (0.2 ± 0.1 μg/mL). Abramson and Clear [19] have suggested that this could be related to differences in the overall sucrose content of each medium: YES (15% sucrose)/CYB (3% sucrose); while CYB may also represent a less hydrophilic layer making it more permeable to lipophilic solvents used in OTA extraction. 

All of the yeast strains tested displayed an ability to completely inhibit OTA contamination in the *Aspergillus* culture filtrate (data not shown). This significant decrease in OTA levels in co-cultures with yeast appears to be related to the inhibition of *A. carbonarius* growth. Nevertheless, OTA production is not necessarily proportional to the biomass of the mycotoxigenic fungi, as it has been shown for other mycotoxins [20]. An increase in OTA production in the fungal biomass may in some instances take place as a consequence of competition among microorganisms for essential environmental factors. Inter-microbial competition is a stressful physiological condition and is known to have a dramatic effect on secondary metabolism in both food spoilage and phytopathogenic fungi. In addition nutrient availability is also known to markedly influence OTA production in other *Aspergilli*. For example OTA production in *A. ochraceus* has been shown to be dependent on nutrient content [21], as well as a variety of nutritional based factors such as various carbon and nitrogen sources [22]. In addition Teren *et al.* [23] have suggested that Aspergilli are capable of assimilating the phenylalanine moiety from the OTA molecule, as a nitrogen source in nutrient replete culture medium.

**Table 1:** Growth of *Aspergillus carbonarius* MPV A566 in YES medium alone or in co-culture with antagonistic *Saccharomyces cerevisiae* or *Kloeckera apiculata* strains after seven days at 25 °C.

Treatment	Colony diameter (cm) ^1^ ± SD		
Experiment	I	II	III
*A. carbonarius*	6.5 ± 0.1	6.3 ± 0.0	6.2 ± 0.0
*A. carbonarius* + *S. cerevisiae* 1090	1.3 ± 0.0 ** ^2^	1.3 ± 0.1 **	1.2 ± 0.1 **
*A. carbonarius* + *S. cerevisiae* 1127	1.5 ± 0.1 **	1.3 ± 0.1 **	1.3 ± 0.0 **
*A. carbonarius* + *S. cerevisiae* 1161	1.3 ± 0.0 **	1.2 ± 0.0 **	1.3 ± 0.1 **
*A. carbonarius* + *S. cerevisiae* 1182	1.2 ± 0.1 **	1.0 ± 0.1 **	1.2 ± 0.0 **
*A. carbonarius* + *S. cerevisiae* 1226	2.0 ± 0.1 **	1.8 ± 0.1 **	2.0 ± 0.0 **
*A. carbonarius* + *Kloeckera apiculata* 3187	3.0 ± 0.1 **	3.1 ± 0.1 **	2.8 ± 0.0 **
*A. carbonarius* + *Kloeckera apiculata* 3197	2.8 ± 0.1 **	2.8 ± 0.1 **	3.0 ± 0.0 **

^1^ Data from three independent experiments are expressed as the colony diameter (cm) after 7 days at 25 °C. ^2^ Values followed by two asterisks are significantly different from the *A. carbonarius* control by Dunnett’s test (*P* < 0.001).

Selected yeast strains were also assessed for their ability to inhibit berry infection by *A. carbonarius* upon co-inoculation. The wine yeast strains significantly reduced fungal colonisation on artificially inoculated grape berries of two cultivars namely Cannonau (a red cultivar) and Vermentino (a white cultivar) (Table 2).

The mean disease reduction rate was up to 70% in all strains tested, ranging from between 80%-99% and 75%-100% for the Vermentino and Cannonau cultivars, respectively (Table 2). Differences in grape varieties are known to affect fungal invasion, with skin hardness and thickness as well as tannin content known to be a hurdle for penetration by the pathogen [24]. *A. carbonarius* is well known to be a very invasive fungus which is capable of colonising and penetrating berries even without skin damage and to grow at 25-35 °C and 0.95-0.99 a_w_, respectively [25]. It should be emphasised that the experimental conditions employed here to assess the potential *in vivo* biocontrol activity of the yeast strains against *A. carbonarius*, were highly favourable to the fungus. OTA accumulation is known to mainly occur at ripening, when the fungus preferentially infects berries by entering skin wounds which are induced either by insects and/or injuries caused by meteorological phenomena. High levels of fungal infection and consequent wine contamination by OTA may then take place when high humidity and temperature conditions occur coupled with grape berry damage. Furthermore, the levels of infection by *A. carbonarius* and the synthesis of OTA are the highest on wounded berries that are detached and that are subject to conducive temperatures, such as those adopted in our laboratory experiments. Thus, although the experimental conditions employed here should have been highly conducive to fungal infection, almost all yeast strains provided an efficient protection to the wine grape berries against infection by *A. carbonarius* for up to seven days (Table 2). Such a time lag could be crucial in ensuring that the wine grape harvest is biologically protected during the most critical phase for OTA contamination, *i.e.*, between harvesting and pressing [3].

**Table 2:** *Aspergillus carbonarius* MPV A566 infection rate on grape berries (cultivars Vermentino and Cannonau) co-inoculated with antagonistic *Saccharomyces cerevisiae* or *Kloeckera apiculata* strains after seven days at 25 °C.

Treatment	Percentage of diseased infected berries ± SD ^1^	
	Vermentino	Cannonau
*A. carbonarius*	100 ± 0.0	100 ± 0.0
*A. carbonarius* + *S. cerevisiae* 1090	0.1 ± 0.1 ** ^2^	0.1 ± 0.1 **
*A. carbonarius* + *S. cerevisiae* 1127	7.1 ± 0.1 **	7.1 ± 0.1 **
*A. carbonarius* + *S. cerevisiae* 1161	6.7 ± 0.1 **	0.1 ± 0.1 **
*A. carbonarius* + *S. cerevisiae* 1182	7.0 ± 0.1 **	0.0 ± 0.0 **
*A. carbonarius* + *S. cerevisiae* 1226	20.1 ± 0.1 **	24.5 ± 4.3 **
*A. carbonarius* + *Kloeckera apiculata* 3187	19.9 ± 0.1 **	0.1 ± 0.1 **
*A. carbonarius* + *Kloeckera apiculata* 3197	0.1 ± 0.1 **	12.9 ± 0.1 **

^1^ Pooled data from three independent experiments carried out on cultivars Vermentino and Cannonau are expressed as mean percent of diseased grape berries of *A. carbonarius* (±SD) after 7 days at 25 °C. ^2^ Values in each column followed by two asterisks are significantly different from the *A. carbonarius* control by Dunnett’s test (*P* < 0.001).

General nutrient competition in the grape berry is not *per se* sufficient to explain yeast biocontrol activity. Nevertheless, this finding does not exclude the possibility that the antagonistic behavior being exhibited by these yeast strains may be as a result of competition for a specific growth limiting factor, such as—for example—a vitamin or another nutrient. According to related pathosystems, other possible mechanisms may include biofilm formation on the wound surface [26], the inhibition of fungal secondary metabolism [27], the production of antifungal enzymes [28] or the induction of resistance in the fruit tissues [12,29]. 

In order to explore possible mechanisms of biocontrol activity the most effective strain, DISAABA1182 was selected for further investigations. In a study involving 20 yeast strains *S. cerevisiae* DISAABA1182 had previously been reported to exhibit the most potent inhibitory effect in decreasing OTA levels [6]. These authors have also shown that as OTA residues were not recovered from the yeast cell biomass and due to the absence of OTα and phenylalanine in the must that another degradation pathway may be employed by this yeast strain. 

The OTA biosynthetic pathway, while as yet not fully elucidated in any fungal species, is believed to involve the synthesis of an isocoumarin group which is a pentaketide formed from an acetate and malonate requiring the involvement of a polyketide synthase (PKS) enzyme. A number of studies have reported a direct link between *pks* gene expression and OTA production [21,22]. In order to examine a possible correlation between reduced OTA production and *pks* gene expression [30,31,32,33] two OTA-producing strains namely *A. carbonarius* MPV A566 and *A. ochraceus* MPV A703 were co-cultured with *S. cerevisiae* DISAABA1182 as well as being exposed to supernatant preparations from the yeast*.*

**Figure 1:**
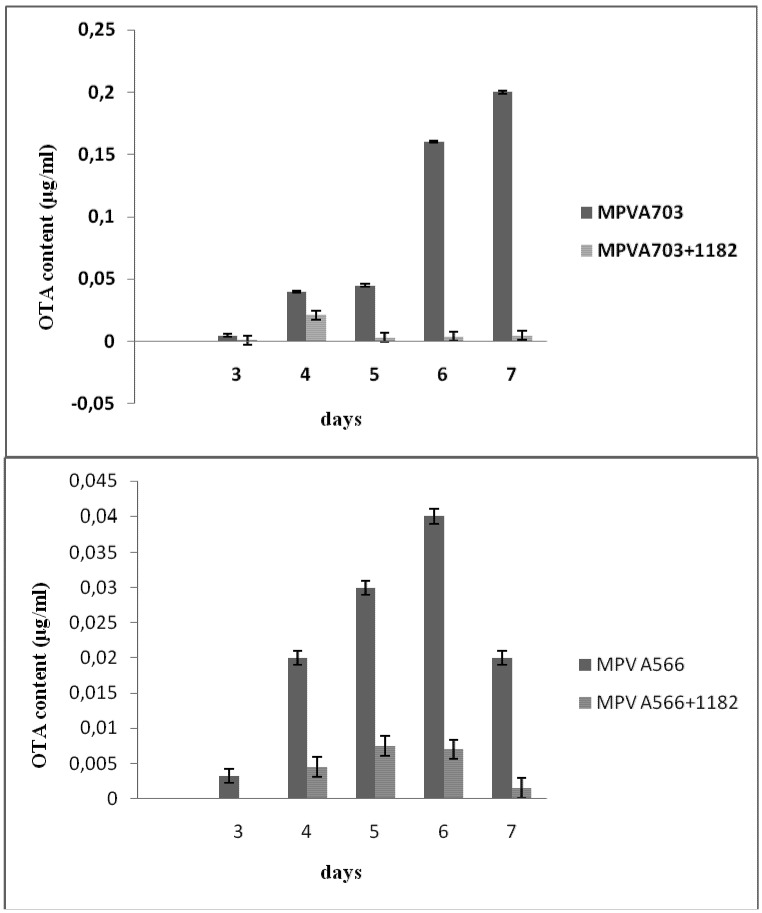
Ochratoxin A (OTA) production by *Aspergillus ochraceus* MPVA703 (upper) and *Aspergillus carbonarius* MPVA566 (lower) grown alone or co-cultured with *S. cerevisiae* DISAABA1182 on YES medium for seven days at 25 °C. OTA values are expressed as μg/mL (±SD).

Following growth of both *A. ochraceus* MPV A703 and *A. carbonarius* MPV A566 under OTA inducing conditions (YES medium), OTA production was observed (Figure 1). With *A. ochraceus* MPV A703, OTA production was first observed on day 3 with levels increasing to reach their highest levels on day 7, while the highest level of OTA produced by *A. carbonarius* MPV A566 was detected on day 6 and started to decrease on day 7 (Figure 1).

When *A. ochraceus* and *A. carbonarius* were grown in co-culture with living cells of *S. cerevisiae* DISAABA1182 living cells, a decrease of 98% and 95% in overall OTA concentrations was observed, respectively. Similarly, a reduction in fungal biomass of up to 95% was observed for both fungi following six days of incubation (Table 3).

To provide further insights into potential mechanisms which may be involved in OTA reduction caused by the yeast, *A. ochraceus* MPV A703 and *A. carbonarius* MPV A566 were statically grown for six days at 25 °C in YES medium amended with either crude supernatant from strain DISAABA1182 or with supernatant which had been previously autoclaved for one hour. A reduction in OTA levels was observed for both fungi with these decreases being accompanied by reductions in fungal biomass (Table 3). Crude supernatant preparations from strain DISAABA1182 reduced OTA production by 99% and 94%, respectively in *A. ochraceus* and *A. carbonarius*; and fungal growth was inhibited by up to 96%. When autoclaved supernatant preparations from DISAABA1182 were tested complete suppression of OTA production in both fungi was observed, while fungal biomass levels were reduced by 58% in the case of *A. ochraceus* and 96% for *A. carbonarius* (Table 3). Thus while in the case of *A. carbonarius* it is quite clear that fungal biomass affects OTA production, *i.e.*, the growth of the fungus corresponds to the production of OTA, this does not seem to apply to *A. ochraceus* when co-cultured with autoclaved supernatant preparations from *S. cerevisiae* DISAABA1182. Schmidt-Heydt and co-workers previously reported that OTA production does not always directly correlate with fungal biomass, and showed that different environmental factors can affect the production of this mycotoxin in different ways [33].

**Table 3:** Fungal growth and OTA production in *A. ochraceus* MPVA703 and *A. carbonarius* MPVA566 grown for six days at 25 °C in YES medium amended with living cells of *S. cerevisiae* strain *DISAABA*1182, together with crude and autoclaved supernatant preparations. Results are expressed as percentage of the untreated control ± SD. Values in each column followed by two asterisks are significantly different from the control as assessed by the Dunnett’s test (*P* < 0.001).

Treatment	*A. ochraceus* MPVA703		*A. carbonarius* MPVA566	
	Fungal growth (%)	OTA (%)	Fungal growth (%)	OTA (%)
Control	100 ± 0.0	100 ± 0.0	100 ± 0.0	100 ± 0.0
*DISAABA*1182 living cells	5.0 ± 0.1 **	2.3 ± 0.1 **	5.6 ± 0.1 **	5 ± 0.1 **
*DISAABA* 1182 crude supernatant	5.1 ± 0.1 **	0.03 ± 0.1 **	5.4 ± 0.1 **	6.3 ± 0.1 **
*DISAABA* 1182 autoclaved supernatant	41.9 ± 0.1 **	0.0 ± 0.1 *	4.2 ± 0.1 **	0.0 ± 0.1 *

**Figure 2:**
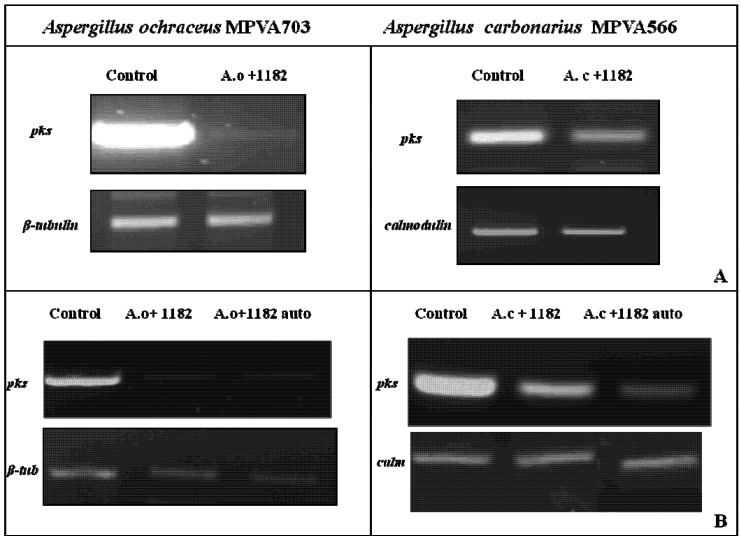
RT-PCR analysis of *pks* gene expression in *Aspergillus ochraceus* MPVA703 and *Aspergillus carbonarius* MPVA566 grown alone or in presence of *Saccharomyces cerevisiae* strain *DISAABA*1182 living cells (**A**), or its crude and autoclaved supernatants (**B**). RNA was extracted from four-day-old mycelium grown statically in YES medium at 25 °C. Reference genes were β-tubulin for *A. ochraceus* and calmodulin for *A. carbonarius*, respectively. Abbreviations: *Aspergillus ochraceus* (A.o); *Aspergillus carbonarius* (A.c); β-tubulin (β-tub); calmodulin (calm).

Thus to determine whether the observed effects were being mediated at the level of gene expression, polyketide synthase (*pks*) gene transcript levels were analysed in *A. ochraceus* and *A. carbonarius* during co-culture with strain DISAABA1182 (Figure 2). Reduced levels of *pks* gene transcript were evident in *A. ochraceus*, while in the case of *A. carbonarius* the *pks* gene expression was only slightly reduced. The expression levels of β-tubulin and calmodulin genes observed under the test conditions suggest that changes in *pks* gene expression are specific and not simply occurring as a result of changes in overall gene transcription levels in the fungus.

As previously mentioned, it has been well established that a number of physiochemical parameters, such as nutritional and environmental stimuli, play an important role in the regulation of mycotoxin biosynthesis [22,34]. Previous studies have shown that OTA production by *A. ochraceus* is dependent on the growth medium [30] and that production is concomitant with increases in *pks* gene transcript levels. A correlation has also been reported between physiological stress factors and the expression of genes responsible for ochratoxin A production in *Penicillium verrucosum* [33]. In our study the strong reduction in *pks* gene expression in *A. carbonarius* and, to a lesser extent, in *A. ochraceus* correlates closely with the reduced OTA levels, suggesting that the observed reduction in OTA production may be mediated at the level of gene transcription (Table 3; Figure 2).

Thus *S. cerevisiae* DISAABA1182 inhibits growth, OTA production and *pks* expression in two ochratoxigenic strains of *A. carbonarius* and *A. ochraceus* and constitutes one of a few reports which have to date described the effects of biocontrol agents on the expression of mycotoxin biosynthetic genes [34,35]. One such report involves an antagonistic strain of *Debaryomyces hansenii* which has been observed to reduce the expression of OTA biosynthetic genes in *A.* *westerdijkiae* [35]. The mechanisms resulting in reduced OTA production and inhibition of *Aspergillus* growth by *S. cerevisiae* DISAABA1182 remain to be further elucidated but are likely to involve a number of synergistic mechanisms, including effects mediated at the level of gene transcription. 

## 3. Experimental Section

### 3.1. Fungal Strains and Culture Condition

The strains *Aspergillus carbonarius* Bainier Thom. MPVA566 and *Aspergillus ochraceus* G. Wilh. MPVA703 belong to the Collection of the University of Piacenza (courtesy Professor Paola Battilani). Spore suspensions of both fungi were prepared by collecting conidia from five-day-old colonies grown on Potato Dextrose Agar (PDA; Merck, Milano, Italy) at 25 °C in distilled water. Spore concentration was determined by using a Thoma haemocytometer and brought to a standard concentration of spores of 10^5^/mL.

Strains of *Saccharomyces cerevisiae* Meyen *ex* Reess and *Kloeckera apiculata* Reess emend. Klöcker belong to the culture collection of University of Sassari-Dipartimento di Agraria. Yeast strains were routinely prepared by inoculating 50 mL of Yeast Peptone Dextrose (YPD), consisting of 1% yeast extract, 2% bacto-peptone (Difco, Franklin Lakes, NJ, USA), 2% dextrose broth (Carlo Erba Reagenti, Milano, Italy), 2% Bacto-agar (Fluka, Milano, Italy), with a loop of cells and by incubating on a rotary shaker (180 rpm) at 25 °C for 24 h. Cell concentration was determined by using a Thoma haemocytometer. The yeast strains were stored in YPD at 4 °C, and in 50% glycerol at −80 °C.

### 3.2. Inhibition of *in Vitro* Growth and OTA Production by *A. carbonarius* and *A. ochraceus*

Inhibition experiments were performed on YPD, CYA (Czapek Yeast Extract) and YES (Yeast Peptone Dextrose; Merck) agar media. Top agar was prepared by mixing 6 mL of medium with 0.7% agar at 40 °C and 1 mL of yeast suspension containing 10^6^ cells/mL. The agar-yeast suspension was poured into Petri dishes that contained 15 mL of agar medium. Once the top agar had set, three 10-μL portions of a spore suspension of *A. carbonarius* MPVA566 (10^5^ CFU/mL) were separately spotted on each plate. Three replicate experiments for each yeast strain were performed, each one consisting of three replicate plates. Plates inoculated with *A. carbonarius* MPVA566 only were used as control. Fungal growth inhibition was determined as the percentage of colony diameter decrease compared to control after 7 days at 25 °C.

In a second set of experiments static cultures of *A. ochraceus* MPVA703 and *A. carbonarius* MPVA566 were grown in YES for 3 days at 25 °C before addition of living cells of *S. cerevisiae* DISAABA1182 (final concentration: 10^6^ cells mL^−1^) and allowed to grown for 8 days under the same conditions.

Static cultures of *A. ochraceus* MPVA703 and *A. carbonarius* MPVA566 were grown in YES amended with supernatant from *S. cerevisiae* 1182 supernatant (cell-free culture filtrate) at a 1:1 ratio of YES:yeast filtrate for 8 days at 25 °C in the dark. *S. cerevisiae* DISAABA1182 culture filtrates were obtained from overnight YES liquid grown cultures (final concentration 10^6^ cells mL^−1^), centrifuged for 20 min at 1500× *g* and filtered through a Millipore 0.22 μm nitrocellulose filter.

Fungal biomass was filtered, thoroughly washed with deionised water, blotted dry with Whatman paper, and stored at −70 °C overnight before freeze-drying.

### 3.3. Effect of Yeast Antagonists on OTA Production by *A. carbonarius*

A co-culture of *A. carbonarius* and each of the tested yeasts strains was grown on liquid CYA and YES media. *A. carbonarius* was also inoculated in yeast-free CYA and YES broth, which was used as control. Following incubation for 7 days at 25 °C in the dark, production of OTA was estimated by High Performance Liquid Chromatography (HPLC), according to Sibanda *et al*. [36] with a Beckman Ultrasphere C18 (250 × 4.6 mm, 5 μM) reversed-phase column. The mobil phase was acetonitrile:water:acetic acid (99:99:2). OTA was detected using a Merck-Hitachi fluorescence detector with an excitation wavelength of 333 nm and an emission wavelength of 460 nm. All samples were diluted 1:1 with HPLC mobile phase prior to analysis.

### 3.4. Biological Control of *A. carbonarius* on Wounded Berries

Mature bunches of grapes of two common Sardinian cultivars, *i.e.*, Cannonau (red) and Vermentino (white), were disinfected with 1% sodium hypochlorite for 10 min and rinsed twice with distilled water. Artificial wounds (2 mm diameter) were made in each berry with a sterile needle to simulate natural damage. Grape bunches were initially dipped in a cell suspension (10^8^ CFU/mL) of each antagonistic yeast strain and then allowed to dry at room temperature before spraying with an aqueous spore suspension of the test fungus (10^4^ CFU/mL) until runoff. Each treatment, consisting of three replicate bunches of grapes (5 berries/bunch), which were placed in plastic containers (60 × 40 × 15 cm, one layer), wrapped in transparent polyethylene bags to prevent evaporation, and stored for 6 days at 25 °C and 85% relative humidity. Positive controls consisted of berries which were treated with sterile water and then sprayed with an *A. carbonarius* MPVA566 spore suspension as described. Three separate experiments were in each case independently conducted. 

### 3.5. Genomic DNA Isolation, RNA Preparation, cDNA Synthesis and RT-PCR

Fungal DNA was extracted according to Al-Samarrai and Schmid [37]. Based on previous *pks* gene expression studies [21,31] mycelium samples were collected at day 4 from *Aspergillus* cultures grown on liquid YES medium. These were filtered, weighted and stored at −70 °C until further use. Stored mycelia was ground to a fine powder in liquid nitrogen with a mortar and pestle. RNA was extracted using a RNasy plant mini kit (Quiagen), treated with DNase I (Roche, Milano, Italy) to remove contaminating DNA and stored at −70 °C. An aliquot of RNA was separated on an agarose gel to check for integrity [38]. The RNA concentration for each sample was determined spectrophotometrically and was in each case brought to an identical value.

cDNA was synthesized from mycelia using reverse transcriptase and random hexamer promoter (Roche) as previously described [39]. The cDNA was used as template for a PCR amplification with a *pks* gene-specific primers pair designed using sequences from O’Callaghan *et al.*, and Gallo *et al.* [21,31] (Table 4). The housekeeping genes calmodulin and β-tubulin from *A. carbonarius* and *A. ochraceus*, respectively (Table 4), were used as a control to monitor expression of constitutively expressed genes.

Amplifications were performed with a GeneAMP system 9600 (Perkin-Elmer) in 25 μL reaction mixture containing: 2.5 μL of *Taq* polymerase buffer 10×, 1 μL of 50 mM MgCl_2_, 1 μL of dNTP 10 mM of each, 1 μM of each primer, 0.5 U of *Taq* (Roche), 50 ng of genomic DNA, H_2_O up to 25 μL. Reaction conditions were: 94 °C for 3 min, then 33 cycles consisting of 94 °C for 1 min, 58 °C for 45 s and 72 °C for 45 s, followed by one final extension step at 72 °C for 10 min. The amplified products were examined by agarose gel electrophoresis following standard methods.

**Table 4:** PCR primers used in the RT-PCR experiments.

Primer name	Sequence
Β-tub F (*A. ochraceus*)	5′-GGCAAACATCTCTGGCGAGCAC-3′
Β-tub R (*A. ochraceus*)	5′-GAAGTTGTCGGGGCGGAAAA-3′
PKS F (*A. ochraceus*)	5′-TCACCTGTCGTATCAGC-3′
PKS R (*A. ochraceus*)	5′-AACTCGGTCAAGCAGATC-3′
Camod F (*A. carbonarius*)	5′-GGCCAGATCACCACCAAG-3′
Camod R (*A. carbonarius*)	5′-TCACGGATCATCGAC-3′
Ac12RL_OTAF (*A. carbonarius*)	5′-AATATATCGACTATCTGGACGAGCG-3′
Ac12RL_OTAR (*A. carbonarius*)	5′-CCCTCTAGCGTCTCCCGAAG-3′

### 3.6. Statistical Analysis

Data from two or three independent experiments were subjected to one-way analysis of variance followed by multiple comparisons by Dunnett’s test, using Minitab^®^ for Windows release 12.1.

## 4. Conclusions

We report here for the first time on the biocontrol activity observed in the wine strain *S. cerevisiae* (DISAABA1182) that decreases growth, OTA production, and *pks* expression in two ochratoxigenic strains of *A. carbonarius* and *A. ochraceus*. This strain displayed good biocontrol characteristics under both in *vitro* and in *vivo* conditions and should in future be considered as an efficient alternative to fungicides to control fungal growth and OTA production on grapes and other fresh or dried fruit commodities which are prone to *Aspergillus* spp. infection and OTA contamination. *S. cerevisiae* DISAABA1182 has previously been shown to be capable of reducing OTA levels in synthetic must [6]; and the present report provides further evidence of the suitability of this strain as a starter in winemaking, particularly with wine grape berries that may be contaminated by OTA. 
